# Supplementary material for: Splice-Junction-Based Mapping of Alternative Isoforms in the Human Proteome
Source: Cell Rep. Author manuscript; Available in PMC 2020 Jan 15. (PMC6961840; doi:10.1016/j.celrep.2019.11.026)
Supplement: 2 [file NIHMS1546469-supplement-2.zip › DF1/PXD010154/Spleen/Spleen_1_TNXB_MGPLSVVIVTVAR.pdf]

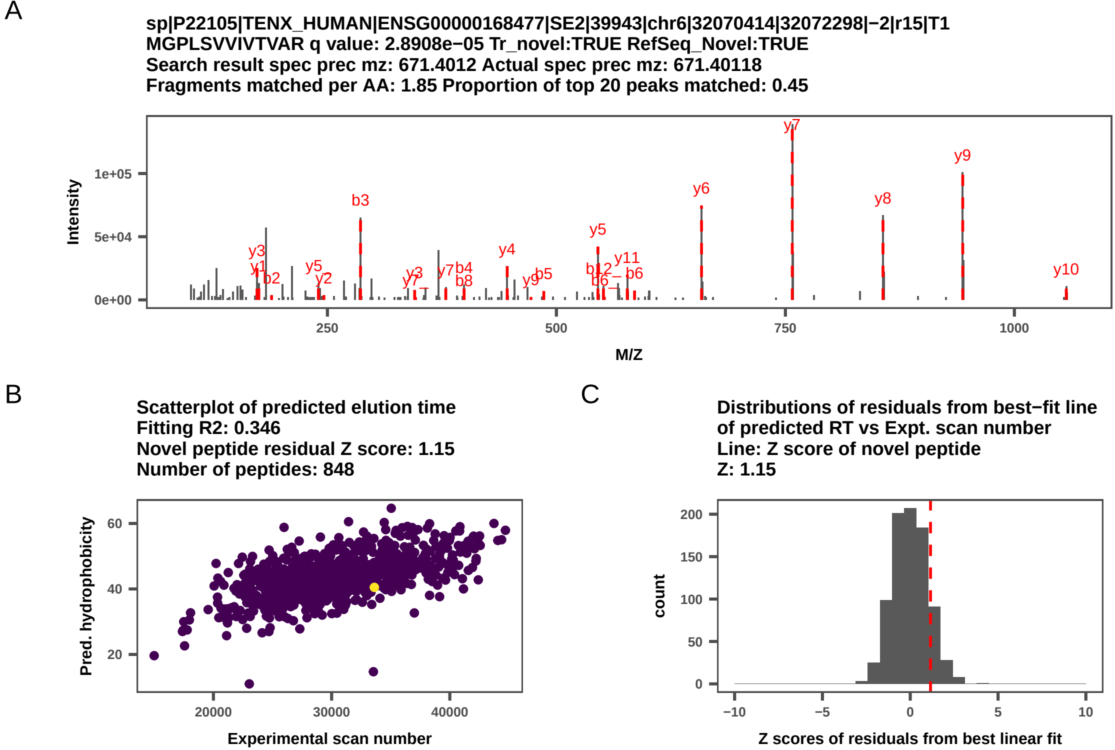
A
sp|P22105|TENX_HUMAN|ENSG00000168477|SE2|39943|chr6|32070414|32072298|−2|r15|T1
MGPLSVVIVTVAR q value: 2.8908e−05 Tr_novel:TRUE RefSeq_Novel:TRUE
Search result spec prec mz: 671.4012 Actual spec prec mz: 671.40118
Fragments matched per AA: 1.85 Proportion of top 20 peaks matched: 0.45
Intensity
1e+05
5e+04
0e+00
M/Z
250
500
750
1000
y7
y9
y6
y8
b3
y5
y4
y11
b6
y10
b5
b8
b2
y2
y3
y1
B
Scatterplot of predicted elution time
Fitting R2: 0.346
Novel peptide residual Z score: 1.15
Number of peptides: 848
Pred. hydrophobicity
60
40
20
20000
30000
40000
Experimental scan number
C
Distributions of residuals from best−fit line
of predicted RT vs Expt. scan number
Line: Z score of novel peptide
Z: 1.15
count
200
150
100
50
0
−10
−5
0
5
10
Z scores of residuals from best linear fit
